# Thelytokous Parthenogenesis in the Fungus-Gardening Ant *Mycocepurus smithii* (Hymenoptera: Formicidae)

**DOI:** 10.1371/journal.pone.0006781

**Published:** 2009-08-26

**Authors:** Christian Rabeling, José Lino-Neto, Simone C. Cappellari, Iracenir A. Dos-Santos, Ulrich G. Mueller, Maurício Bacci

**Affiliations:** 1 Section of Integrative Biology, The University of Texas at Austin, Austin, Texas, United States of America; 2 Departamento de Biologia Geral, Universidade Federal de Viçosa, Viçosa, Minas Gerais, Brazil; 3 Center for the Study of Social Insects, São Paulo State University, Rio Claro, São Paulo, Brazil; Field Museum of Natural History, United States of America

## Abstract

The general prevalence of sexual reproduction over asexual reproduction among organisms testifies to the evolutionary benefits of recombination, such as accelerated adaptation to changing environments and elimination of deleterious mutations. Documented instances of asexual reproduction in groups otherwise dominated by sexual reproduction challenge evolutionary biologists to understand the special circumstances that might confer an advantage to asexual reproductive strategies. Here we report one such instance of asexual reproduction in the ants. We present evidence for obligate thelytoky in the asexual fungus-gardening ant, *Mycocepurus smithii*, in which queens produce female offspring from unfertilized eggs, workers are sterile, and males appear to be completely absent. Obligate thelytoky is implicated by reproductive physiology of queens, lack of males, absence of mating behavior, and natural history observations. An obligate thelytoky hypothesis is further supported by the absence of evidence indicating sexual reproduction or genetic recombination across the species' extensive distribution range (Mexico-Argentina). Potential conflicting evidence for sexual reproduction in this species derives from three *Mycocepurus* males reported in the literature, previously regarded as possible males of *M. smithii*. However, we show here that these specimens represent males of the congeneric species *M. obsoletus*, and not males of *M. smithii*. *Mycocepurus smithii* is unique among ants and among eusocial Hymenoptera, in that males seem to be completely absent and only queens (and not workers) produce diploid offspring via thelytoky. Because colonies consisting only of females can be propagated consecutively in the laboratory, *M. smithii* could be an adequate study organism a) to test hypotheses of the population-genetic advantages and disadvantages of asexual reproduction in a social organism and b) inform kin conflict theory.

For a Portuguese translation of the abstract, please see Abstract S1.

## Introduction

Explaining the prevalence of sexual over asexual reproduction has been a longstanding goal in evolutionary biology [1]–[5]. Over 20 models describing the advantages of sex have been proposed, differing with respect to the short- and long-term advantages of sexuality that outweigh its two-fold evolutionary cost [2], [6]–[8]. The most generally applicable models postulate fitness benefits for recombining organisms, either because of faster adaptation to changing conditions (e.g., the Red-Queen model) [9], [10], or because of more efficient purging of deleterious mutations (mutation-based models) [11], [12]. The near absence of asexual lineages that persist over long evolutionary time suggests that the disadvantages of asexuality generally outweigh its benefits [13], [14]. However, asexual organisms arise sporadically throughout the tree of life, and the study of obligate long-term asexual organisms may hold the answer to why almost all eukaryotes reproduce via meiosis and syngamy.

Asexuality is part of normal hymenopteran reproduction because males are produced via arrhenotoky (males develop from unfertilized, haploid eggs) [15], [16]. In contrast, females develop from fertilized, diploid eggs [17], [18]. Thelytokous parthenogenesis, the production of diploid female offspring from unfertilized eggs, has been observed in a small number of eusocial hymenopteran species. So far, thelytoky has been convincingly demonstrated in the Cape honeybee, *Apis mellifera capensis* [19]–[21], and for seven distantly related species of ants (see Discussion).

*Mycocepurus smithii* is a common, but cryptic species of fungus-gardening ants, which is widely distributed throughout Latin America and obligately relies on a symbiotic fungus for food [22]–[24]. Recently, independent lines of evidence indicated that *M. smithii,* might be capable of thelytokous reproduction. First, the absence of males in three years of continuous fieldwork in Puerto Rico led Fernández-Marín et al. [25] to hypothesize that queens of *Mycocepurus smithii* might reproduce via thelytoky. A comparative study of *Mycocepurus* populations in Brazil found that in contrast to *M. goeldii*, *M. smithii* nest entrances occurred in high densities and that nests seemed to be connected to each other, forming a colony network [26], [27]. The connected nest systems of *M. smithii* suggested that colonies multiply by fission, potentially influenced by the reproductive biology of the species [26], [27]. Additionally, Himler et al. [28] observed thelytoky of *M. smithii* in laboratory colonies from Panama. Males were not encountered in *M. smithii* nests during the *Mycocepurus* mating season in Peru, Brazil (Rabeling, pers. obs.), Puerto Rico [25] and Panama [28], even though sympatric, sexually reproducing *Mycocepurus* species engaged in nuptial flights, suggesting that *M. smithii* might be obligately asexual.

The existence of three unidentified *Mycocepurus* males collected by W. E. Kerr in Rio Claro, São Paulo State in Brazil [29] appear to challenge the hypothesis that *M. smithii* is obligately asexual. Until recently, only two *Mycocepurus* species were known from São Paulo State: *M. goeldii* and *M. smithii*. The males of *M. goeldii* had been previously described [30], and hence, by process of elimination, the males collected by Kerr were tentatively assigned to *M. smithii*. Interestingly, Kempf [22] described the morphology of these males in his taxonomic revision of the genus, but had the foresight to not name them, because the specimens were unassociated with a maternal colony, making it impossible to identify them with confidence. The ‘Kerr males’ therefore could belong to an undescribed species or to a described species not yet known from São Paulo State.

In order to test if thelytoky is widespread in queens of *M. smithii* and to investigate the behavioral ecological consequences of thelytoky in field populations, we studied the population biology, reproductive physiology of queens and workers, and the mating behavior of a *M. smithii* population in Brazil, and compared this population with two sexually reproducing, sympatric congeners. In addition, we provide taxonomic evidence for the identity of the ‘Kerr males’.

## Materials and Methods

### Population Biology of *Mycocepurus smithii*

Fieldwork was conducted from September 29^th^ to October 26^th^ 2008 on the Campus of São Paulo State University (UNESP) in Rio Claro, Brazil (22.3955°S, 047.5424°W; elevation 608 m). In the State of São Paulo, the nuptial flights of most attine ant species, including those of *Mycocepurus* [29], occur during late September and October, which mark the beginning of the rainy season. In order to maximize the chance of finding males in the nests of *M. smithii*, we focused collecting on the probable mating season.

**Figure 1:**
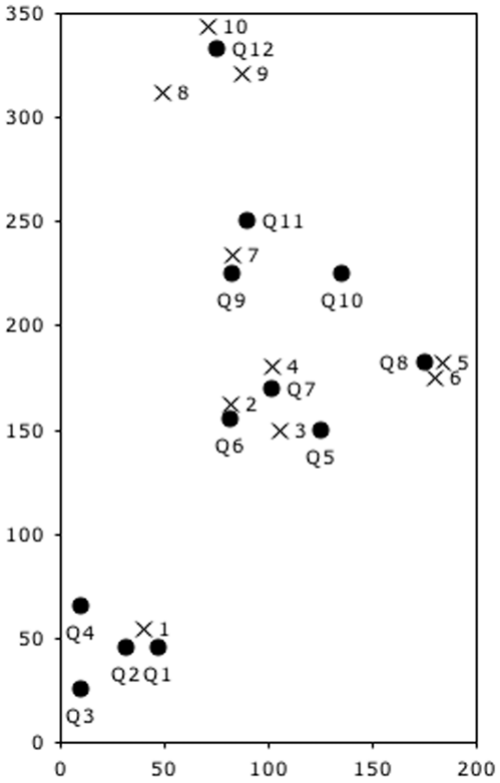
Spatial distribution of nest entrances and fungus chambers of the studied *Mycocepurus smithii* population. Nest entrances are depicted as Xs, queenright chambers as solid black dots and all other fungus chambers as open black circles; size of circles does not represent the relative sizes of nest chambers. The x and y axes are labeled in cm. The nest excavation was continued 50 cm beyond the study plot limits in all four directions and no additional chambers were found.

Previous studies demonstrated that it might be difficult to recognize *M. smithii* nests as separate units because nest entrances may occur in high densities and lateral tunnels connect underground chambers [27]. We therefore selected a study site where nest entrances were spatially separated from other *M. smithii* populations and an entire nest aggregation could be studied through excavation, including all reproductively active and potentially inseminated queens. Once the target aggregation was identified, the study area was marked and the nest entrances mapped, resulting in a 3.5 m×2 m study area with 10 nest entrances (Fig. 1). Throughout the excavation process, the study plot was frequently sprayed with water to increase the visibility of nest entrances (the ants open the nest entrances after the entrance hole has been closed by washed-in soil). These freshly excavated soil mounds are easier to recognize than older nest entrances, which sometimes erode over time during periods of drought. A pilot study conducted at the same site in 2006 demonstrated that *M. smithii* nests do not occur in layers deeper that 70 cm (Rabeling, unpublished). In order to excavate and survey all underground chambers of the entire aggregation, a trench of 150 cm depth was dug outside the marked study plot at a distance of at least 50 cm from the first nest entrance. Vertical slivers of soil were then sliced away with a sharpened spade. The position of all nest chambers was recorded in a grid documenting the spatial distribution within the study population. The digging was continued 50 cm past the limits of the study area.

Colonies of *Mycocepurus goeldii* and an undescribed *Mycocepurus* species served as a positive control for sexually reproducing species. Nests of *M. goeldii* were shown to have a single nest entrance [27]. Single, spatially separated colonies were chosen and a trench of 250 cm depth was dug in 50 cm distance from the first nest entrance, since pilot study showed that *M. goeldii* chambers could be encountered as deep as 190 cm below the surface (Rabeling, unpublished). Vertical slivers of soil were sliced away with a spade and the position of all nest chambers were also recorded in a grid. The digging was continued 50 cm past the last excavated chamber.

All *Mycocepurus* specimens were stored in 95% ethanol for further examination and censusing. Voucher specimens of this study were deposited in the entomology collection of Maurício Bacci's Molecular Evolution Laboratory at São Paulo State University (UNESP), the Museum of Comparative Zoology at Harvard University (MCZ), the Museu de Zoologia da USP (MZUSP), and in C. Rabeling's collection.

To supplement the results of the *M. smithii* nest excavations and provide direct evidence for polydomy, different nest casting methods were tried, following methodologies described by Williams and Lofgren [31] and Tschinkel [32]–[34]. However, the tunnel diameter of *M. smithii* nests was as narrow as 1 mm, which did not permit plaster of Paris or molten zinc to trace the tunnels. Other casting techniques will be required in the future to illustrate that nests of *M. smithii* are connected via tunnels.

### Reproductive Biology of *Mycocepurus* queens

All *Mycocepurus* specimens were collected in 95% ethanol and dissected in 0.2 M phosphate buffer. Female reproductive tracts were examined using an Olympus SZ40 dissecting scope and an Olympus CX31 microscope. Photographs of the female reproductive tracts were taken with a Nikon D200 SLR digital camera mounted on an Olympus SZH10 dissecting scope or an Olympus CX31 microscope. To test if queens were inseminated, the spermathecae were dissected, opened and examined under a microscope, to ensure that the content were sperm and no other kind of proteinaceous secretion. The developmental status of the ovaries was recorded as undeveloped (0), developing without fully developed oocytes (1) and fully developed ovaries with mature oocytes present (2) (Table 1). The presence of yellow bodies (corpora lutea) at the base of the ovaries was also recorded. Yellow bodies are remnants of follicular cells and indicate if the respective female oviposited in the past. In case of a parthenogenetic, reproductively active female, one would expect an empty spermatheca, fully developed ovaries with mature oocytes, and corpora lutea as an indicator of oviposition activity in the past. Reproductively inactive females could have both empty and sperm-filled spermathecae, depending on insemination status, but their ovaries are expected to be undeveloped with no yellow bodies. In contrast, a sexually reproducing female would have a sperm filled spermatheca, developed ovaries, and yellow bodies.

**Table 1:** Reproductive status of dissected *Mycocepurus* queens.

species	n	spermatheca	ovary development (*)	corpora lutea
*M. smithii* (dealate)	12	translucent/empty	2	present
*M. goeldii* (dealate)	2	opaque w/sperm	2	present
*M. goeldii* (alate)	5	translucent/empty	0	absent
*M*. sp. nov. (dealate)	15	opaque w/sperm	1 (n = 12); 2 (n = 3)	absent (n = 12); present (n = 3)
*M*. sp. nov. (alate)	2	translucent/empty	0	absent

(*) The state of ovary development was divided in three categories: 0 = not developed, 1 = developing but without fully developed eggs, 2 = well developed with mature eggs present. In nests of *M*. sp. nov. more than one queen was found in a single chamber. Among those queens some were reproductively active, while others were inseminated, but the ovaries were still developing. The presence of corpora lutea indicates if a female oviposited.

To test if queens were the only reproductively active individuals in the colonies a total of 50 workers were dissected. Five workers were randomly chosen from five queenright (chambers 4, 30, 37, 45 & 59; see Table S1) and five queenless chambers (chambers 13, 21, 42, 47 & 50; see Table S1) and dissected following the methodology described above.

### Behavioral observations of nuptial flights

Under thelytoky and in the absence of males, the mating behavior is expected to degenerate, including the behavioral preparations of workers when modifying nest entrances for mating flights. Preliminary behavioral observations in previous years showed that the sexually reproducing *Mycocepurus goeldii* modifies its nest mounds in preparation of the nuptial flights of the alates (see results for details). These modified nest entrances serve as a proxy if a particular colony is preparing for a nuptial flight. Between September 29^th^ and October 15^th^ the nest entrances of ten *M. goeldii* colonies were monitored three times per day and compared to the ten entrances of *M. smithii* in the study plot.

### Taxonomic identity of the ‘Kerr males’

For most *Mycocepurus* species only the worker caste is described. To find males that match the ‘Kerr males’, special emphasis was given to collecting workers, queens, and males from any *Mycocepurus* colonies. *Mycocepurus obsoletus* is one of the species known only from workers and we collected entire colonies in a Cerrado habitat at the margin of a gallery forest at the Reserva Ecológica do IBGE, 35 km south of Brasília, in the Federal District of Brazil (15.9436°S, 047.8767°W; elevation 1087 m). To collect reproductives, the entrances of several nests were flagged during the dry season in July 2008 and on November 12^th^ 2008, with the first rains of the season, alate *Mycocepurus* males (n = 19) and queens (n = 13) were collected next to the previously marked nests. The external morphology of the males was compared to the ‘Kerr males’ under a Leica Z6 APO dissecting scope.

Additionally, these males were identified to species using molecular sequence information to ensure the males from Brasília are in fact *Mycocepurus obsoletus* males. The partial DNA of three single-copy nuclear markers (Elongation Factor 1-alpha F1 copy, Wingless and Long Wavelength Rhodopsin) and one mitochondrial gene (Cytochrome Oxidase I) were sequenced, resulting in 3512 base pairs, and compared to sequence information from *M. obsoletus* workers collected at the same site. The resulting molecular phylogeny will be presented in the context of an ongoing generic revision (C. Rabeling in prep.).

## Results

### Population Biology of *Mycocepurus smithii*

A total of ten *M. smithii* nest entrances were identified in the 7 m^2^ study plot (1.43 entrances/m^2^) (Fig. 1). The excavation of this population revealed a total of 59 fungus chambers (see Table S1 for exact chamber contents). The entire population consisted of 851 workers and 12 queens, averaging approximately 14 workers per chamber and one queen in every 5^th^ fungus chamber (see Table S1). The maximum number of workers per chamber was 43, the minimum one. All queenright chambers contained only a single reproductively active queen, however, the number of queens encountered was higher than the number of nest entrances in the study area. The brood was distributed in 21 (36%) chambers and consisted of 115 early instar larvae, 40 worker pupae, 51 queen larvae and 274 queen pupae. The sexual brood was restricted to ten chambers (17%) and in eight of them worker brood and sexual brood co-occurred. The maximum and minimum number of sexual brood per chamber was 80 and one, respectively. No male pupae and no alate queens and males were present in the nest chambers.

Because of the close proximity of some of the nest entrances (Fig. 1), it was not possible to assign some chambers to a single nest entrance. However, the chambers were distributed in clusters close to the nest entrances. Nest entrance #1 was located 116 cm distant to the next entrance (#3) with 15 fungus chambers underneath, four of them queenright. Under entrances 2, 3 and 4 a total of 15 chambers was encountered, three of them queenright. Six fungus chambers were located under entrances 5 and 6, of which one contained a queen and entrance number 7 possibly led to nine fungus chambers, two of them containing a reproductively active queen. Twelve fungus chambers were associated with entrances 8, 9 and 10, of which a single chamber contained a queen. Two chambers, one of them queenright (Fig. 1), were almost equidistant to entrances 4, 5/6 and 7 and no entrance was encountered above these chambers.

The spatial distribution of queenright chambers and nest entrances were not paired, indicating that some of the colonies have more than one reproductively active queen, while others use more than one entrance to access their nest. The fungus chambers were distributed in depths between 10 and 66 cm below the surface, well above the depth of the excavation pit of 150 cm depth throughout the study plot.

The spatial distribution of *M. goeldii* entrances and queenright chambers were exactly paired. Each of the two nests excavated had a single nest entrance, and each nest was recognizable as single spatially separate units, containing five or six fungus chambers, respectively. Both colonies had a single reproductively active queen.

### Reproductive Biology of *Mycocepurus* queens

**Figure 2:**
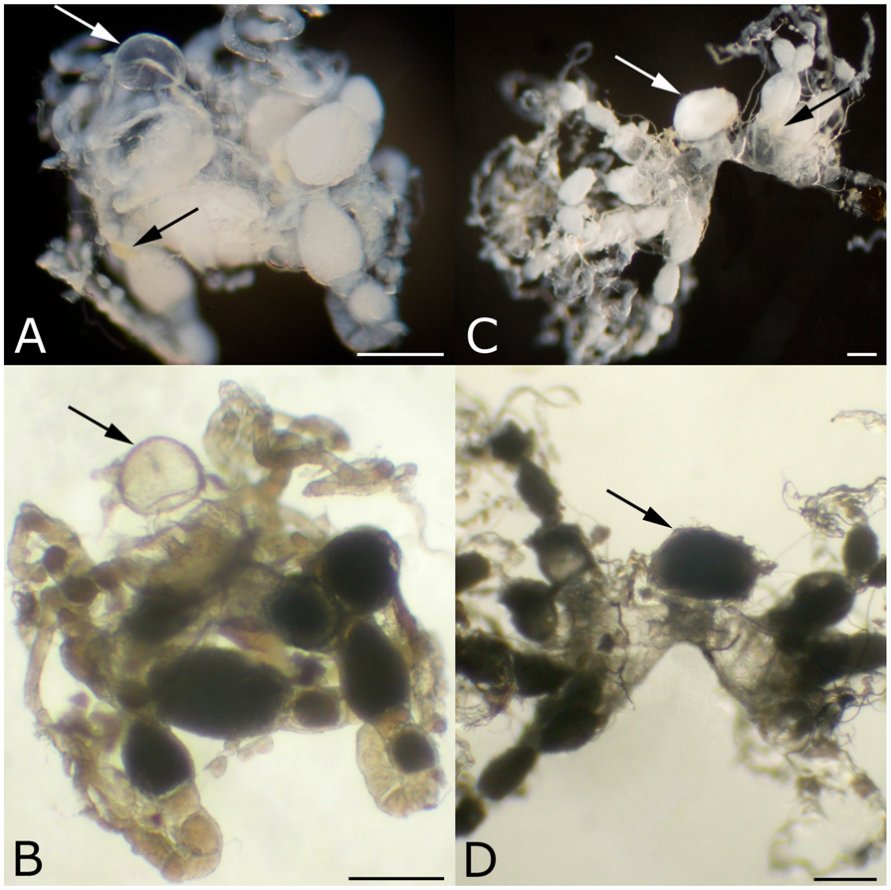
Internal reproductive organs of *Mycocepurus smithii* (A, B) and *M. goeldii* queens (C, D) in reflecting light (A, C) and transmission light (B, D). Arrows drawn from the top left to the bottom right point to the spermatheca and arrows drawn from the top right to the bottom left point to yellow bodies. (A, B) The spermatheca of *M. smithii* is translucent (empty), the ovaries are developed and yellow bodies are present. (C, D) A reproductively active *M. goeldii* queen has an opaque, sperm-filled spermatheca, developed ovaries and yellow bodies. The scale bar represents 2 mm in all images.

Since spermatheca and ovary morphology are variable among different ant genera, we dissected the reproductive tracts of 36 *Mycocepurus* queens (*M. smithii,* n = 12; *M. goeldii*, n = 7; *M*. sp. nov., n = 17; see Table 1). Dealate and alate queens of *M. goeldii* and an undescribed *Mycocepurus* species served as positive controls for inseminated queens and as negative controls for non-inseminated females, respectively. Twelve queens of *M. smithii* had translucent spermathecae, not revealing any content upon opening. The ovaries of these females were well developed and contained several mature oocytes per individual. The base of each ovariole had multiple corpora lutea, indicating the reproductive activity of each female (Fig. 2). Hence, all *M. smithii* queens showed the signs of thelytokous reproduction: empty spermathecae, developed ovaries, and presence of corpora lutea confirming past oviposition. In contrast, the two dealate *M. goeldii* queens both had opaque spermathecae with sperm content. The ovaries of both queens were well developed with several mature oocytes present and the base of each ovariole contained multiple corpora lutea, confirming reproductive activity of these females. Five alate, virgin *M. goeldii* queens had empty spermathecae, undeveloped ovaries with short ovarioles, which did not contain differentiated premature oocytes, and yellow bodies were absent. The queens of *M*. sp. nov. could be divided in three reproductive categories. First, dealate, reproductively active queens (n = 3) had sperm-filled spermathecae, developed ovaries containing mature oocytes and corpora lutea. Second, dealate, inseminated queens (n = 12), which did not have fully developed ovaries and lacked yellow bodies. Instead the ovarioles of each ovary were in the process of development, and did not contain mature oocytes, but premature oocytes were present. Third, alate, virgin queens (n = 2), which had empty spermathecae, undeveloped ovaries and absent corpora lutea.

The examined queens of *M. goeldii* and *M*. sp. nov. confirmed the predictions for sexually reproducing species: virgin, non inseminated queens had undeveloped ovaries and the absence of corpora lutea indicated that the respective female never oviposited. In contrast, the reproductively active females had sperm-filled spermathecae, developed ovaries and the presence of corpora lutea indicated oviposition. Some dealate queens, which were not reproductively active (indicated by the absence of corpora lutea), were also inseminated and had developing ovaries.

None of the 50 examined *M. smithii* workers was reproductively active. Each worker had a pair of ovaries with a single ovariole per ovary. Ovarioles did not contain mature oocytes and yellow bodies were absent. All workers were lacking a spermatheca.

### Behavioral observations of nuptial flights

Nest observations started on September 29^th^ in Rio Claro, before the onset of the rainy season. At that time, nest entrances of *M. goeldii* and *M. smithii* consisted of loose, irregularly shaped soil mounds of 5–20 cm diameter, each with a single entrance. Rains started on October 2^nd^, stimulating *M. goeldii* workers to increase the number of nest entrances to about 20–30 entrances per nest mound. Consequently, the soil mound surrounding the entrances had a sponge like appearance. Several *M. goeldii* workers waving their antennae surrounded each entrance hole. Upon modification of the mound, males of *M. goeldii* could be observed moving in the passages of the sponge like nest mound, although the males did not surface. Queens were not observed. On October 3^rd^, cooler weather and rain showers caused temperatures to drop by 5–10°C. During this period, the nest entrances of *M. goeldii* stayed open or were reopened after rain had washed soil into the entrance tunnels. The workers of *M. goeldii* were still surrounding the entrance holes and alate males and queens could be seen in the nest mound tunnels. On the morning of October 7^th^, the temperature had increased to about 25°C, and the winds and the rain had stopped. At approximately 10.00 h, the males of *M. goeldii* colonies started the nuptial flight. First resting on the rim of each entrance crater, then spreading the wings and flying up vertically, perhaps gathering in the lower branches of a tree, as observed by Kerr [29]. The alate queens started flying slightly later at 11.30 h. The release of alates from each colony continued until 15.00 h. During the rest of the afternoon, workers closed the supernumerary nest entrances, until each colony had only a single entrance.

During this same time period, and until the end of the study three weeks later, the entrances of *M. smithii* nests remained unmodified, with some soil surrounding a single entrance hole. In addition, neither mating flights, nor modified nest mounds, nor males were ever observed throughout several years of field research in Latin America (Rabeling, field observations 2003–2008).

### Taxonomic identity of the ‘Kerr males’

**Figure 3:**
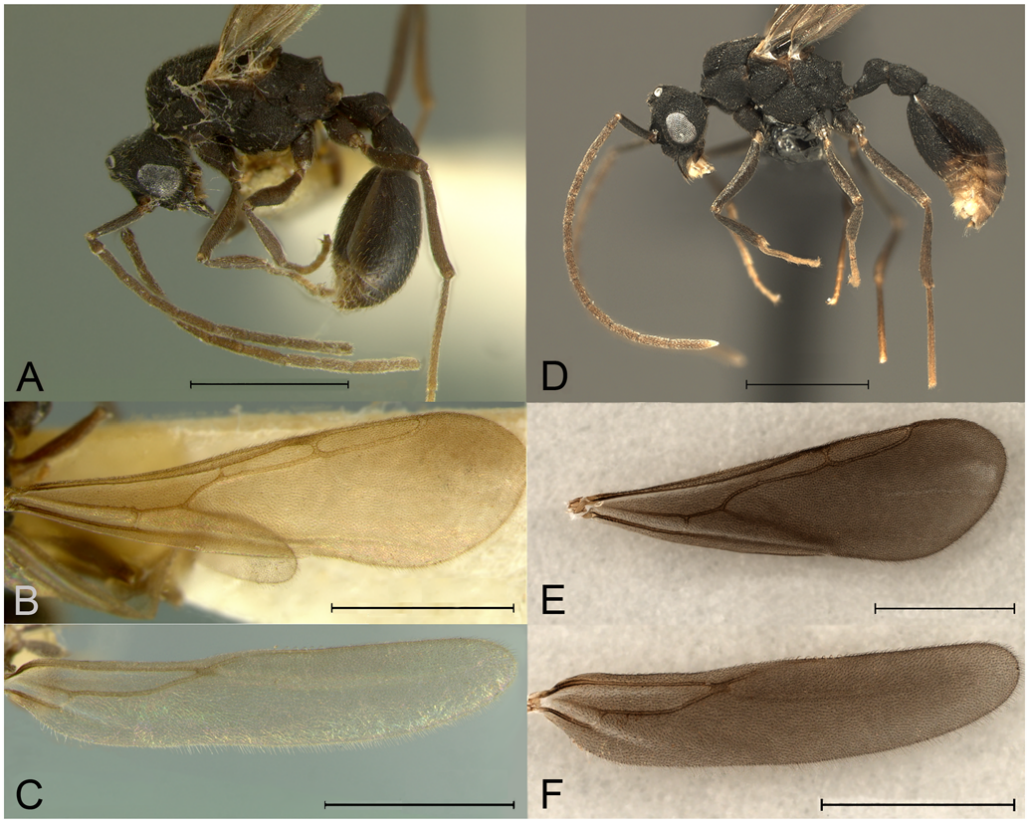
Morphological comparison of one of the *Mycocepurus* males collected by W. Kerr in Rio Claro in 1960 (A, B, C) and a *Mycocepurus obsoletus* male (D, E, F). (A) lateral view of ‘Kerr male’, (B) forewing and (C) hindwing in dorsal view. (D) Male of *Mycocepurus obsoletus* in lateral view, (E) forewing and (F) hindwing in dorsal view. Morphological characters informative for species identification are the shape of the propodeal spine, of the petiole and the wing venation. Scale bars represents 1 mm in all images.

On November 12^th^, 19 males and 13 queens were collected when exiting the marked colonies of *Mycocepurus obsoletus*. The previous day, the first heavy rainstorm of the season soaked the soil for approximately 24 hours and stopped at 10.00 h of November 12^th^. Two hours later (12.00 h) alate males and queens were observed to lift off from the leaf litter for 10–50 cm before landing again in the litter. The nuptial flight lasted for approximately two hours from noon to 14.00 h and was interrupted by a brief rain shower; after the rain shower (15.00 h), no activity could be detected at the marked nests. It is unclear whether alates flew into the surrounding vegetation and formed mating aggregations, as has been reported for *M. goeldii* [29], or mated in the leaf litter. The morphological comparison of the alate males to the males collected by Kerr in 1960 revealed that males from both collections, Brasília and Rio Claro, are almost identical (Fig. 3). Identifying characters for *Mycocepurus* males include the shape of the propodeal spine, the petiole and the wing venation (Fig. 3). Given the morphological variability observed within and between all species of *Mycocepurus* (Rabeling in prep), the ‘Kerr males’ appear to be specimens of *M. obsoletus*. A comparison of the male sequences to sequences of *M. obsoletus* workers from the same locality resulted in 100% sequence identity, confirming the identity of those males. Consequently, the ‘Kerr males’ are the males of *M. obsoletus* and not the males of *M. smithii*.

## Discussion

Several lines of direct evidence (queen reproductive physiology) and indirect evidence (taxonomy & natural history) implicate obligate thelytokous parthenogenesis for a Brazilian population of the fungus-gardening ant species *M. smithii*. The presence of mature oocytes and yellow bodies indicate that dissected queens had oviposited and were reproductively active, whereas the empty spermathecae prove that queens were not inseminated. In combination, these characteristics indicate thelytokous parthenogenetic propagation and support Fernández-Marín’s [25] hypothesis of asexuality in *M. smithii*, formulated to explain the absence of males in a Puerto Rican *M. smithii* population.

**Figure 4:**
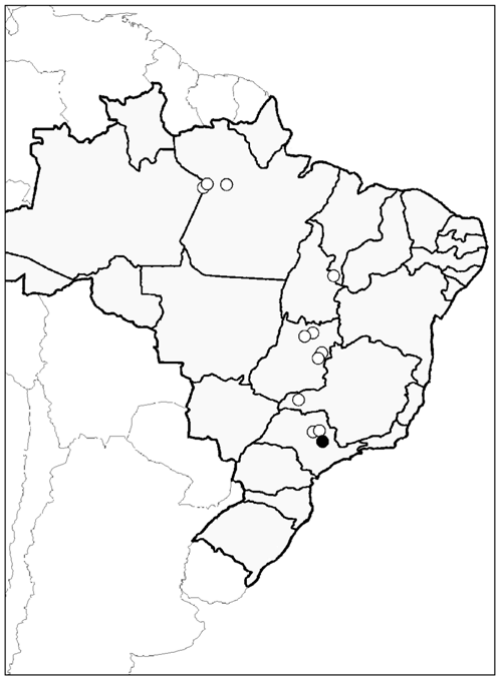
Geographic distribution of *Mycocepurus obsoletus*. Thick black lines depict the boundaries of Brazilian States, open circles represent collections based on worker specimens, the solid black dot depicts the collection site of the ‘Kerr males’: Rio Claro in São Paulo Sate. From north to south, *M. obsoletus* has been recorded in the States of Pará, Tocantins, Goiás, the Federal District, Minas Gerais and São Paulo. Besides Rio Claro, *M. obsoletus* has been collected in two other localities in São Paulo Sate: a) Reserva Ecológica de Jataí near Luis Antônio (23–25.V.1997, Silvestre & Silva col.; see [35]), which is located approximately 100 km north of Rio Claro, and b) Ibitinga (25.I.1964, K. Lenko col.), which is approximately 150 km northwest of Rio Claro.

Three males from Rio Claro in Brazil [22], [29] represented a challenge to the obligate asexuality hypothesis, because they were suspected to be males of *M. smithii*, indicating the potential for sex and recombination in this species. Our morphological study, however, indicates that the ‘Kerr males’ are the previously unknown males of *M. obsoletus,* not males of *M. smithii*. Previously, *M. obsoletus* was not known to occur in Rio Claro, but we now know that *M. obsoletus* has a Brazilian Cerrado distribution (Fig. 4), thus its occurrence in the Rio Claro area is certainly possible. In addition, *M. obsoletus* workers have been collected at two other relatively nearby localities in São Paulo State (Fig. 4; see also [35]), further increasing the likelihood of its occurrence at or near Rio Claro. Thus the ‘Kerr males’ seem to represent only the southernmost presently known distribution record of *M. obsoletus* (Fig. 4).

Matching the ‘Kerr males’ to *M. obsoletus* provides only circumstantial evidence for obligate thelytoky, but it does remove the strongest potential counter evidence, i.e.: that the ‘Kerr males’ are actual males of *M. smithii*. While we cannot rule out the possibility that *M. obsoletus* and *M. smithii* males are morphologically identical; this is most unlikely, however, given the observed morphological variation within and between *Mycocepurus* species (Rabeling, pers. obs.).

Within eusocial Hymenoptera, arrhenotokous parthenogenesis is a widely distributed phenomenon [15], [36] and the reproductive modes in ants are particularly diverse [37]. In contrast, thelytokous parthenogenesis, the production of female offspring from unfertilized eggs, has been rarely confirmed in eusocial Hymenoptera, although the number of recognized thelytokous species is increasing. So far, thelytoky has been demonstrated convincingly for the South African honey bee subspecies *Apis mellifera capensis* [19]–[21] and seven ant species, which can be divided into three categories of asexual reproduction: queens produce queens and workers via thelytoky, but workers are sterile and males seem to be absent (called here: **type A** thelytoky); workers produce workers and potentially queens via thelytoky (**type B**); or queens produce queens thelytokously and workers sexually, and workers produce queens thelytokously (**type C**). Males are known for all type B and type C asexual ant lineages, suggesting the potential for sex and recombination, but males remain unknown for type A asexuals, of which *M. smithii* is the first known example in the Formicidae.

Among asexual ant species, worker thelytoky (**type B** thelytoky) occurs in four distantly related ant species, the cerapachyine ant *Cerapachys biroi* [38]–[40], the two myrmicine ants *Messor capitatus* [41] and *Pristomyrmex punctatus* [42]–[48], and the ponerine ant *Platythyrea punctata* [49]–[52]. The thelytokous production of queens by queens and sexual production of workers by queens (**type C**) has so far been documented in the formicine ant *Cataglyphis cursor* [53]–[56], and the two myrmicines *Wasmannia auropunctata* [57]–[59] and *Vollenhovia emeryi* [60]–[61].

At present, *Mycocepurus smithii* seems to be the only representative of **type A** asexual ant species. Natural history studies in Puerto Rico [25] and the Brazilian Amazon [26], [27] suggested an unusual reproductive biology for *M. smithii*. Fernández-Marín et al. [25] first proposed the hypothesis that *M. smithii* might reproduce thelytokously. This study shows that *M. smithii* queens from the Rio Claro, Brazil population are thelytokous. Himler et al. [28] dissected six queens from a Panamanian population, reaching the same conclusion. Based on these three geographic data points, Brazil, Panama, both confirmed, and Puerto Rico, still unconfirmed, it seems that thelytoky is widespread across *M. smithii*'s distribution range.

Firm evidence capable of identifying the cytological mechanism of thelytoky (i.e., apomixis versus automixis) in *M. smithii* is currently lacking. A comprehensive genetic population study in combination with cytological evidence is necessary to distinguish between meiotic and mitotic parthenogenesis. Himler et al. [28] tested queens and workers of 12 Panamanian colonies for allelic variation at a single variable genetic locus and found queens and offspring to be genetically identical, concluding that *M. smithii* propagates clonally. However, during automixis with central fusion, two haploid nuclei are produced during the first meiotic division, which subsequently fuse to form the diploid zygote. Gene recombination does not necessarily occur and thus, the genetic signature of apomicticly and automicticly produced offspring can be very similar if not identical [15], [56], [62], [63]. A single genetic locus is hence insufficient to distinguish between mitotic and meiotic parthenogenesis.

*Wolbachia* bacteria induce parthenogenetic reproduction in some parasitoid wasps [64]. Several hymenopteran species have been tested for *Wolbachia* infections, but *Wolbachia* could never be demonstrated to induce parthenogenesis in any thelytokous eusocial Hymenoptera species [28], [41], [52], [65]. This supports the hypothesis that a sex determining system based on heterozygosity may form a limitation to *Wolbachia*-induced parthenogenesis [65]. *Wolbachia* endosymbionts induce the production of homozygous diploid offspring through gamete duplication [66], [67], which results in the exclusive production of diploid males. In ants and eusocial bees, females are required to be heterozygous at the sex-determining locus, a condition difficult to achieve when parasitized by a gamete duplicating endosymbiont [21], [65]. Because thelytoky seems to have evolved independently of *Wolbachia* in ants and bees, it also seems unlikely that thelytoky of *M. smithii* queens was induced by *Wolbachia*. The absence of *Wolbachia* in *M. smithii* supports this expectation and additional antibiotic treatment could not ‘cure’ *M. smithii* from parthenogenesis [28].

The wide geographic distribution could suggest an ancient origin of asexuality in *M. smithii*. Low variability in DNA sequence, however, does not support an ancient asexuality hypothesis, but rather argues for a recent origin of thelytoky followed by a rapid population expansion (Rabeling, unpublished). The widespread geographic distribution also suggests that geographic parthenogenesis, the occurrence of parthenogenetically reproducing lineages towards the margin of a distribution range [68], [69], unlikely applies to *M. smithii*, because both São Paulo State and Panama are located well within the continuous distribution range, which extends from Northern Mexico to Argentina [22,23;Rabeling, in prep].

Theory predicts that morphological structures degenerate if not maintained by natural selection. Panamanian *M. smithii* queens apparently lack the so-called mussel organ, and Himler et al. [28] therefore argued for degenerate mating apparatus, which is thought to support an ancient origin of asexuality in *M. smithii*. The mussel organ is a sclerotized structure in the reproductive tract of leaf cutter ant queens, which is thought to protect the female genitalia tissue from damage inflicted by sclerotized hooks of the male's penis [70]. In an obligately asexual species both the spermatheca and the mussel organ are expected to lose their function. Given that soft tissues like the spermatheca were present and not reduced in size in *M. smithii* queens when compared to two sexual congeners, it seems premature to argue for ancient asexuality based on the absence (or non-detection) of the mussel organ. Ideally, a comparative functional-morphological study should first establish a) whether the mussel organ is present in closely related, sexually reproducing *Mycocepurus* species, because *Mycocepurus* and *Atta* shared the most recent common ancestor some 50 Million years ago and are therefore only distantly related to each other [71]; b) whether the mussel organ in *M. smithii* is homologous to the respective structure in *A. colombica* for which this organ was first described [70]; and c) to what degree sclerotization varies with age, because weakly sclerotized structures are often translucent in recently eclosed arthropods.

High relatedness is expected in colonies of *M. smithii* whether queens reproduce via apomictic or automictic thelytoky. Kin conflict theory predicts conflict between individual kin or groups of kin (i.e., castes) within a colony, because individuals or castes differ in relatedness, reproductive success or both [72]. In a society of highly related or even clonal organisms kin conflict over reproduction is expected to be reduced if not entirely absent. However, Hamilton's rule [73], [74] does not account for low frequency mutations with strong selective effects and therefore, relatedness alone is not always a good predictor of kin conflict over reproduction [75], [76]. Once the genetic underpinnings of reproduction in *M. smithii* societies are understood, this ant should be an interesting study organism for kin selection theory.

Behaviors specific to sexual reproduction might degenerate rapidly in parthenogenetic lineages [77]. In *M. smithii*, modifications of the nest related to nuptial flight activity were never observed during six field-seasons (2003–2008), even though alate queens were present in *M. smithii* nests. At the same time sympatric sexual species showed modified nest structures and performed nuptial flights. The observation that *M. smithii* queens were still in the pupal stage whereas sexually reproducing species performed nuptial flights suggests that *M. smithii* queens may be produced over a wider time interval than in sympatric, sexual congeners. In the absence of males, it is unnecessary for *M. smithii* queens to depart in a synchronized population wide nuptial flight. Consequently, it appears unnecessary to produce young queens in synchrony and to open the nest entrance for mating flights, which could possibly increase the risk of predation and parasitism.

Alternative to a nuptial flight, young queens could establish themselves in a new chamber that is part of the maternal colony. Such colony expansion would be consistent with the observation that multiple reproductively active *M. smithii* queens inhabit the same colony [25], [27]. However, the ability of independent nest foundation seems to persist in *M. smithii*, as infrequently alate *M. smithii* queens can be found in the leaf litter (Rabeling, pers. obs.) and Fernández-Marín et al. [25], [78] observed claustral nest founding of single dealate queens in Puerto Rican *M. smithii*.

Asexual lineages are capable of colonizing new habitats and exploiting new food sources more efficiently than sexually reproducing relatives [2], [4], [5]. Interestingly, *M. smithii* relies on multiple, genetically distinct lineages of fungi for food [26], [79], [80]. Surveys of fungal diversity cultivated by attine ants suggested that the majority of ants are associated with one fungal lineage [79] and in the case of *Cyphomyrmex* ants, the existence of two distinct fungal cultivar clades revealed the presence of two cryptic ant species [81]. However, this trend does not necessarily apply to all species, as was demonstrated by a comprehensive survey of microbial diversity associated with the North American attine species, *Trachymyrmex septentrionalis* [82]. This study identified four genetically distinct fungal lineages or species in 74 *T. septentrionalis* nests. For *M. smithii*, fewer samples were screened (n = 20), but these few samples already revealed twice the number of genetically distinct cultivars, which moreover spanned almost the entire diversity of lower attine cultivars [26], [79]. No firm conclusion can be drawn from these preliminary results, but the observed pattern is consistent with the hypothesis that asexuals exploit new food sources effectively and colonize new habitats rapidly. A similar effect can be observed in cyclical parthenogenetic animals like water fleas (*Daphnia*), aphids, flukes or some gall wasps living in seasonal environments: during the summer, food sources are abundant and reproduction switches to parthenogenesis, whereas later in the season, when resources become scarce, populations adopt sexual reproduction [4, pp. 57–62]. Because *M. smithii* inhabits both tropical and subtropical habitats, the need for sex to persist in seasonal environments may not be pressing, but a comprehensive comparison of *Mycocepurus* cultivars grown by asexual and sexual hosts will have to test this hypothesis more thoroughly.

Several naturalists have discussed the possibility that *M. smithii* colonies are polydomous, i.e., single colonies occupy more than one nest [22], [25], [27], [83], [84]. Conflicting reports stem from frequent observations of the patchy distribution and high nest densities characteristic of *M. smithii* populations. Small areas often contain multiple nest entrances, as much as six entrances per square meter [26], whereas adjacent areas may have none at all. So far, only two studies have investigated the nest architecture systematically. Fernández-Marín et al. [25] concluded that *M. smithii* colonies had a single nest entrance in Puerto Rico, whereas Rabeling et al. [27] found that in the Brazilian Amazon large nest systems were interconnected through lateral tunnels, potentially enabling the exchange of workers and brood, which would argue for polydomy. In the Rio Claro *M. smithii* population, some nest chamber clustered underneath 2 or 3 spatially separated nest entrances (6 chambers below entrances #5 and #6 and 12 chambers below entrances #8, #9 and #10; Fig. 1) and cluster contained only a single reproductively active queen. In addition, a queenright chamber was located in equal distance to four nest entrances (#4, #5/6 and #7; Fig. 1) and this chamber had no detectable entrance above it. These patterns suggest that nests are connected underground and workers move freely between chambers, sharing the same entrances, further supporting polydomy. The alternative hypothesis could be that additional, potentially inseminated queens of the population escaped during excavation and that some entrances remained inactive and thus remained undetected during the study period of a month. Because of high levels of relatedness are expected for a strictly thelytokous species, it might be that *M. smithii* might not only be polydomous but also unicolonial.

Eidmann [85] first hypothesized that *M. smithii* colonies were polygynous, because “in every of the numerous examined fungus chambers” he found “several old, dealate queens” (p. 86). Unfortunately, Eidmann never tested his hypothesis by dissecting the queens. Fernández-Marín et al. [25] further substantiated Eidmann's hypothesis, suggesting that *M. smithii* colonies might be secondarily polygynous, because pleometrosis, the foundation of colonies by multiple queens, does occur in some colonies. Dissections confirmed that multiple queens from the same nest were reproductively active, as indicated by the presence of developed ovaries and yellow bodies. However, it remains unclear if the dissected queens were found in the same fungus chamber or if they could have been reproductive females from different colonies. In addition, Amazonian *M. smithii* colonies had several dealate queens in the same fungus chamber, but their reproductive status was not confirmed through dissections [27].

In the Rio Claro population, only a single queen was found per fungus chamber (n = 10). The presence of several queenright chambers in one fungus chamber cluster below a single entrance suggests that one polydomous colony can contain multiple reproductively active queens, supporting functional polygyny. Other clusters of fungus chamber, however, inhabited only a single reproductively active queen. In case these clusters are not part of the same polydomous nest, the Rio Claro *M. smithii* population would comprise a mosaic of monogynous and polygynous colonies, which would be consistent with Fernández-Marín et al. 's [25] hypothesis of secondary polygyny. Again, a genetic population study will have to test if queens and worker offspring share the same genetic signature or if multiple mothers produced the workers of a single colony.

### Conclusions

Three types of thelytokous reproduction have been documented in eight distantly related ant species. *Mycocepurus smithii* appears to be obligately asexual based on its reproductive physiology, and the absence of males and mating behavior. A population genetic study of *M. smithii* should show if this fungus-gardening ant species relies exclusively on thelytoky or if cryptic sex does occur. *Wolbachia* endosymbionts seem unlikely as parthenogenesis-inducing agent in eusocial Hymenoptera in general. Ancient asexuality might be suggested by the wide geographic distribution, but rendered unlikely by presence of fully developed soft tissue (i.e., spermatheca) in the mating apparatus of *M. smithii* queens and low genetic variability between populations. For all thelytokous ant species we lack the kind of evolutionary ecology framework that might indicate how long asexuality has persisted in the respective species and what factors might have influenced the rise of thelytokous populations from a sexually reproducing ancestor. The fact that *M. smithii* cultivates multiple lineages of fungi is consistent with the hypothesis that asexuals are efficient colonizers of novel habitats and food sources. Due to the expected high relatedness within *M. smithii* colonies this species is likely to inform kin conflict theory. Careful natural history observations and behavioral experiments coupled with population genetic and phylogenetic studies will certainly answer some of these questions and further increase the already broad spectrum of reproductive diversity in eusocial Hymenoptera and beyond.

## Supporting Information

Abstract S1 (0.03 MB DOC)

Table S1 (0.16 MB DOC)
