# Supplementary material for: Thelytokous Parthenogenesis in the Fungus-Gardening Ant Mycocepurus smithii (Hymenoptera: Formicidae)
Source: PLoS One. 2009 Aug 26;4(8):e6781. doi: 10.1371/journal.pone.0006781 (PMC2728836; doi:10.1371/journal.pone.0006781)
Supplement: Abstract S1 — (0.03 MB DOC) [file pone.0006781.s001.doc]

**Thelytokous parthenogenesis in the fungus-gardening ant *Mycocepurus smithii* (Hymenoptera: Formicidae)**

Christian Rabeling^1^*, José Lino-Neto^2^, Simone Cappellari^1^, Iracenir A. Dos-Santos^2^, Ulrich G. Mueller^1^ & Maurício Bacci Jr.^3^

**Resumo**

A predominância de reprodução sexuada sobre a assexuada comprova os benefícios da recombinação, tais como: adaptação rápida em ambientes sob constantes mudanças e eliminação de mutações deletérias. Registros de reprodução assexuada em organismos que normalmente se reproduzem de forma sexuada desafiam evolucionistas à entender quais circunstâncias podem favorecer estratégias reprodutivas assexuadas. Neste estudo, nós documentamos um caso de reprodução assexuada em formigas, apresentado evidências de telitoquia obrigatória em uma espécie assexuada de formiga cultivadora de fungos, *Mycocepurus smithii*. Nesta espécie as rainhas produzem fêmeas a partir de ovos não fertilizados, operárias são estéreis e machos parecem ser inexistentes. Diagnosticamos a telitoquia obrigatória através de dados fisiológicos da rainha, ausência de machos, ausência de comportamento de acasalamento e observações de história natural. A hipótese de telitoquia obrigatória é ainda corroborada pela ausência de evidências que indiquem reprodução sexual ou recombinação genética em populações desta espécie ao longo de toda sua ocorrência (do México até a Argentina). Registros prévios na literatura de machos de *Mycocepurus* eram tidos como contra-hipótese de uma possível reprodução assexuada nesta espécie, já que estes espécimes foram por muito tempo considerados machos de *M. smithii*. Contudo, demonstramos aqui que estes machos pertencem à espécie congenérica, *M. obsoletus*. De fato, *M.smithii* é a única espécie de Hymenoptera social sem indivíduos do sexo masculino e também é a única em que a produção de cria por telitoquia é feita somente por rainhas (e não por operárias). Em virtude da facilidade com que colônias de fêmeas de *M. smithii* podem ser propagadas em laboratório, esta espécie representa um modelo adequado para estudar as vantagens e desvantagens da reprodução assexuada, em organismos sociais sob os aspectos da genética de populações, bem como gerar conhecimentos sobre a teoria de conflito de parentesco.
