# Supplementary material for: Thelytokous Parthenogenesis in the Fungus-Gardening Ant Mycocepurus smithii (Hymenoptera: Formicidae)
Source: PLoS One. 2009 Aug 26;4(8):e6781. doi: 10.1371/journal.pone.0006781 (PMC2728836; doi:10.1371/journal.pone.0006781)
Supplement: Table S1 — (0.16 MB DOC) [file pone.0006781.s002.doc]

**Table S1**: Census of studied *Mycocepurus smithii* population. All 59 chambers contained a fungus garden. (*) In early larval stages, worker and queen larvae cannot be differentiated based on size alone; therefore, the numbers presented under the category “larvae” could be of either caste. Queen larvae are recognized by their above worker size.

| **chamber** | **workers** | **queens** | **larvae*** | **worker pupae** | **queen larvae** | **queen pupae** | **collection-ID** |
| --- | --- | --- | --- | --- | --- | --- | --- |
| 1 | **10** | - | - | - | - | - | CR081001-02 |
| 2 | **2** | - | - | - | - | - | CR081001-03 |
| 3 | **17** | **1** | - | - | - | - | CR081001-04 |
| 4 | **28** | **1** | - | - | - | - | CR081001-05 |
| 5 | **2** | - | - | - | - | - | CR081001-06 |
| 6 | **19** | - | - | - | - | - | CR081001-07 |
| 7 | **9** | - | - | - | - | - | CR081001-08 |
| 8 | **8** | - | - | - | - | - | CR081001-09 |
| 9 | **22** | - | **1** | **2** | - | - | CR081001-10 |
| 10 | **5** | - | - | - | - | - | CR081001-11 |
| 11 | **10** | **1** | **3** | **8** | - | - | CR081001-12 |
| 12 | **16** | **1** | **10** | **2** | - | - | CR081010-01 |
| 13 | **28** | - | **3** | **8** | - | - | CR081010-02 |
| 14 | **5** | - | - | - | - | - | CR081010-03 |
| 15 | **4** | - | - | - | - | - | CR081010-04 |
| 16 | **18** | **1** | **11** | **11** | - | - | CR081010-05 |
| 17 | **8** | - | - | - | - | - | CR081010-06 |
| 18 | **20** | - | - | - | - | - | CR081011-01 |
| 19 | **9** | - | - | - | - | - | CR081011-02 |
| 20 | **30** | - | - | - | - | - | CR081011-03 |
| 21 | **30** | - | - | - | - | - | CR081011-04 |
| 22 | **16** | - | **2** | - | - | - | CR081011-05 |
| 23 | **7** | **1** | - | - | - | - | CR081011-06 |
| 24 | **7** | - | - | - | - | - | CR081011-07 |
| 25 | **14** | - | - | - | - | - | CR081011-08 |
| 26 | **10** | - | **2** | - | - | - | CR081011-09 |
| 27 | **1** | - | - | - | - | - | CR081011-10 |
| 28 | **3** | - | - | - | - | - | CR081011-11 |
| 29 | **2** | - | - | - | - | - | CR081011-12 |
| 30 | **23** | **1** | **6** | - | - | - | CR081012-01 |
| 31 | **6** | - | - | **8** | **11** | **8** | CR081012-02 |
| 32 | **11** | - | **3** | - | - | - | CR081012-03 |
| 33 | **5** | - | - | - | - | - | CR081012-04 |
| 34 | **9** | - | - | - | - | - | CR081012-05 |
| 35 | **4** | - | - | - | - | - | CR081012-06 |
| 36 | **19** | **1** | - | - | - | - | CR081012-07 |
| 37 | **28** | **1** | - | - | - | - | CR081016-01 |
| 38 | **10** | **1** | - | - | - | - | CR081016-02 |
| 39 | **17** | - | **5** | - | **2** | - | CR081016-03 |
| 40 | **14** | - | - | - | - | - | CR081017-01 |
| 41 | **6** | - | - | - | - | - | CR081017-02 |
| 42 | **30** | - | **5** | - | - | - | CR081017-03 |
| 43 | **21** | - | - | - | - | - | CR081017-04 |
| 44 | **23** | - | - | - | - | - | CR081017-05 |
| 45 | **23** | **1** | **1** | - | **1** | - | CR081017-06 |
| 46 | **10** | - | - | - | - | - | CR081017-07 |
| 47 | **32** | - | - | - | - | - | CR081017-08 |
| 48 | **16** | - | - | - | - | - | CR081018-01 |
| 49 | **1** | - | - | - | - | - | CR081018-02 |
| 50 | **43** | - | **16** | - | **20** | **58** | CR081018-03 |
| 51 | **19** | - | **1** | - | **6** | **64** | CR081018-04 |
| 52 | **22** | - | - | - | **6** | **51** | CR081018-05 |
| 53 | **25** | - | **1** | - | **3** | **77** | CR081018-06 |
| 54 | **9** | - | - | - | - | - | CR081018-07 |
| 55 | **3** | - | - | - | **1** | **1** | CR081018-08 |
| 56 | **16** | - | **7** | - | - | - | CR081018-09 |
| 57 | **7** | - | **6** | **1** | **1** | **13** | CR081018-10 |
| 58 | **17** | - | - | - | - | - | CR081018-11 |
| 59 | **22** | **1** | **32** | - | - | **2** | CR081018-12 |
| **Total** | **851** | **12** | **115** | **40** | **51** | **274** |  |
| **Mean** | **14.42** | **0.2** | **3.59** | **1.74** | **1.96** | **10.96** |  |
| **Count** | **59** | **12** | **18** | **7** | **9** | **8** |  |
